# Nonpapillary prone endoscopic combined intrarenal surgery: effectiveness, safety and tips, and tricks

**DOI:** 10.1007/s00345-022-04178-x

**Published:** 2022-10-17

**Authors:** Panagiotis Kallidonis, Arman Tsaturyan, Gabriel Faria-Costa, Begona Ballesta Martinez, Angelis Peteinaris, Constantinos Adamou, Konstantinos Pagonis, Anastasios Natsos, Theofanis Vrettos, Evangelos Liatsikos

**Affiliations:** 1 Department of Urology, University of Patras Medical School, University of Patras, 26500 Rio, Patras, Greece; 2 Department of Urology, Unidade Local de Saúde de Matosinhos, Matosinhos, Portugal; 3 Department of Surgery and Physiology, Faculty of Medicine of the University of Porto, Porto, Portugal; 4 Department of Anesthesiology and ICU, University of Patras, Patras, Greece; 5 Department of Urology, Medical University of Vienna, Vienna, Austria

**Keywords:** Nonpapillary PCNL, ECIRS, Intrarenal surgery, Indications, Tips and tricks

## Abstract

**Purpose:**

To evaluate the effectiveness and safety of nonpapillary prone endoscopic combined intrarenal surgery (ECIRS) and provide practical tips and tricks for the successful accomplishment of the procedure respecting the anatomical particularities.

**Material and methods:**

This study is an analysis of a prospectively collected database including all cases of ECIRS performed between January 2019 and December 2021 in a high-volume tertiary center. All patients underwent the procedure in prone-split leg position. A nonpapillary renal puncture was performed. The used access sheaths were 22Fr or 30Fr. Lithotripsy was performed anterogradely with a dual-energy lithotripter with incorporated suction and retrogradely with holmium Yttrium–Aluminum–Garnet laser.

**Results:**

A total of 33 patients were included. The initial stone-free rate (SFR) was 84.8% and the final SFR was 90.9%. The median stone size was 35 mm and 60% of patients had staghorn calculi. The prevalence of renal abnormalities was 21.3%, including 3 cases of horseshoe kidney, 2 cases of malrotation and 2 cases with complete duplicated systems. The median operative time was 47 min. The median hospital stay was 3 days and median hemoglobin loss was 1.2 gr/dL. Overall, the complication rate was 9.1%, all being Grade II complications (*n* = 2 fever and *n* = 1 transient bleeding).

**Conclusions:**

Nonpapillary prone ECIRS is an effective and safe procedure. Standardization of the procedure is critical to achieve good outcomes. Patients who benefit the most are probably the ones where additional punctures can be avoided using this technique, namely patients with renal abnormalities, incrusted ureteral stents and staghorn stones.

## Introduction

Endoscopic combined intrarenal surgery (ECIRS) was firstly described by Scoffone et al. in 2008 [1]. It refers to a combination of PCNL with retrograde flexible ureterorenoscopy (fURS). The main objective of combining both procedures is to achieve higher stone-free rates (SFR) after a single procedure. In their series, Scoffone et al. reported that in 33% of the cases, the URS was fundamental to detect calyceal stones inaccessible by PCNL and treat ureteral stones. The initial reported SFR was 81.9% [1], increasing to 90% as reported 10 years later by the same authors [2]. Other studies have also reported on the efficacy and safety of ECIRS, with the most recent systematic review demonstrating a SFR between 61 and 97% and complication rates between 5.8 and 44%, with most being grades I–II according to Clavien–Dindo classification [3]. Furthermore, when compared with PCNL alone, ECIRS has higher SFRs, lower complications, less need for multiple punctures, less fluoroscopy time, and shorter hospital stay while maintaining similar operative time [3].

Over the years there has been increasing interest in ECIRS and different groups shared important technical aspects, crucial to obtain better outcomes [3–5]. Variations of the originally described technique have emerged such as the use of mini-PCNL in ECIRS (mini-ECIRS) [5, 6]. However, the technical aspect that has gathered more discussion has been patient positioning: Galdakao-modified supine Valdivia (GMSV) vs prone split-leg position. In the initial description of the ECIRS technique [1], the GMSV position was interpreted as crucial for good coordination. However, different studies succeeded to demonstrate the feasibility of ECIRS in a prone split-leg position [5, 7–9]. Although outcomes seem to be comparable, there is still a lack of studies for prone ECIRS affirmation as a reproducible technique.

Since 2017, our group has also unveiled another unspoked topic, the nonpapillary puncture in PCNL. We have shown the safety of nonpapillary puncture in a retrospective study [10], a randomized controlled trial [11] and we have also shown that the areas of parenchyma access with either papillary or nonpapillary punctures are equally vascularized [12].

The current article aims to evaluate the effectiveness and safety of nonpapillary prone ECIRS. In addition, the paper aims to provide practical tips and tricks for the successful accomplishment of the procedure respecting the anatomical particularities.

## Methodology

### Study design and study population

A database of all ECIRS procedures in our department was prospectively collected between January 2019 and December 2021. All patients undergoing the procedure were included in this study. No exclusion criteria were applied.

### Surgical technique

**Fig. 1:**
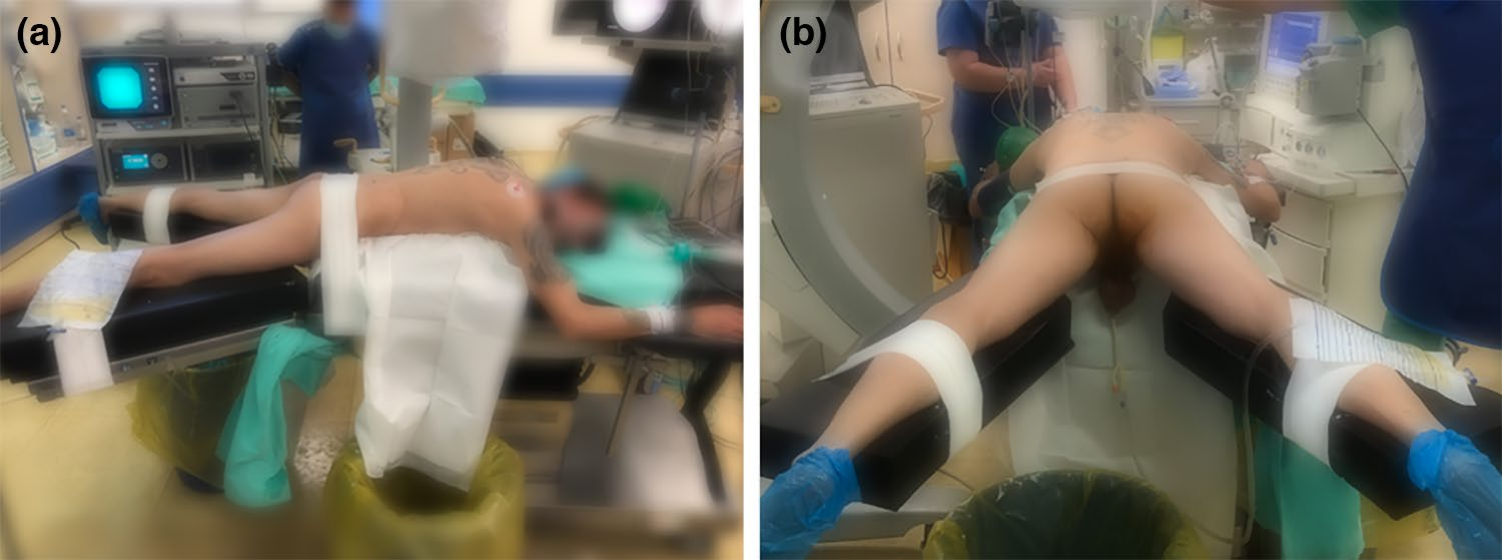
Prone split-leg position. In male patients it is important to keep free access to the penis for retrograde access

Every procedure was performed under general anesthesia. All procedures were performed by the same two surgeons, who accounted for high expertise in both PCNL and RIRS. The patients were either submitted to prior ureteral stent placement—prestented—or were given alfa-blockers 1 week prior to the surgery. In the beginning of the procedure patients were positioned in lithotomy and an open-end ureteral catheter was inserted through a rigid cystoscope. A retrograde pyelogram was obtained, and the ureteral catheter was secured at the level of the ureteropelvic junction. The patient was then turned to the prone split-leg position (see Fig. 1).

A nonpapillary renal puncture was performed and tract dilation was made with Amplatz dilators (Amplatz renal dilator set, COOK Medical, Bloomington, Indiana, USA). An one-step or 2-step technique was used to introduce a 22Fr or 30Fr access sheath, respectively. A 18Fr rigid nephroscope was used with the 22Fr access sheath and a 26Fr scope was used with the 30Fr access sheath. Technical details of both puncture and tract dilation have been described previously [10]. A dual lumen ureteral catheter was retrogradely introduced, and a second guidewire was placed. A ureteral access sheath (Flexor, COOK Medical, Bloomington, Indiana, USA) was placed above the working stiff guidewire. If the patient had a previous ureteral stent the size of the UAS was 12/14Fr. For nonprestented patients, an 9.5/11.5Fr UAS was used. A flexible ureterorenoscope (Flex-XC 11278VS®, Karl Storz, Tuttlingen, Germany with 12/14Fr UAS or PU3033A, PUSEN Medical, Shenzhen, China with 9.5/11.5Fr UAS) was then progressed through the access sheath and a pyeloscopy was performed. Kidney stones were preferentially fragmented with Lithoclast Trilogy® (EMS Medical, Nyon, Switzerland). Whenever stones could not be reached with the nephroscope, retrograde lithotripsy was performed with a holmium laser Cyber Ho 150® (Quanta System, Samarate, Italy) or MOSES Pulse 120H (Lumenis Ltd, Yokneam, Israel) devices.

Energy settings varied between 1 and 2 J with the frequency ranging from 30 to 60 Hz, according to a previously described “self-popping” technique [13]. Nitinol baskets were used in some cases to relocate and present fragments for subsequent removal through the antegrade percutaneous access.

At the end of the procedure, a final retrograde nephroscopy was performed to identify the presence of remaining stones. A double J stent (6–8Fr) and either a malecot tale tubes (20–24Fr) or a balloon nephrostomy tube (16–18Fr) were placed. At day 2–3 postoperatively, nephrostomy/malecot tube was removed and patients were discharged. The Double-J stents were removed between 2 and 4 weeks after the procedure. The operative time was considered from the start of the puncture until the placement of the nephrostomy.

#### Stone assessment, perioperative management and follow-up

Stone characteristics were assessed by imaging study before the procedure. The stone size was considered to be the maximum diameter reported by the radiologist. Stones were defined as complete staghorn calculi if the renal pelvis, upper, middle, and lower calyces were embedded. Partial staghorn was considered as a stone which filled the renal pelvis and one of the calyces.

Infection prevention was performed with a single intravenous dose of either a fluoroquinolone or an aminoglycoside.

A blood test was performed on the first day postoperatively to determine hemoglobin loss, among other parameters. One month after surgery, a kidney–ureter–bladder (KUB) plain radiography and ultrasonography (US) were performed to assess residual stones. If the patients were symptomatic or abnormal findings were found on KUB or US a noncontrast-enhanced computer tomography (NCCT) was performed. In total, 39.4% of the patients (*n* = 13) underwent a NCCT.

### Study variables and statistical analysis

The following data were collected: patient demographic (age, gender and body mass index), stone characteristics (total stone size and presence of partial/complete staghorn calculi), operative data (operative time, number and size of antegrade accesses, stone-free rate and complications). The following outcomes were evaluated: stone-free rate (defined as the absence of any stone at KUB, US or NCCT performed 1 month after the procedure); the need for a second-look PCNL; hospital staying days and complications according to the Clavien–Dindo classification [14] (bleeding: assessed by hemoglobin drop at the first day postoperatively; fever: defined as body temperatures of > 38.0 °C).

Descriptive statistical analyses were performed using SPSS v. 21.0 (SPSS Inc., 2012). Median values and interquartile ranges (IQR) were used for continuous variables and proportions were employed for categorical ones.

## Results

A total of 33 patients were treated with prone nonpapillary ECIRS during the analyzed period. Patient demographics, stone, and perioperative characteristics are summarized in Table 1. The median age was 54 (IQR 49–67) years old with a median body mass index of 25.6 (IQR 23.2–29.0). The median stone size was 35.0 (IQR 28.5–43.5) mm. Partial or complete staghorn calculi were present in 60.1% of the cases. In total, 29 patients were prestented and the remaining 4 patients were receiving alfa-blockers. Two patients had an extensive encrusted stent in the proximal loop. The median operative time was 47 (IQR 36–65) minutes. Most stones were treated with only one puncture, while two and three accesses were required in 15.2% and 6.1%, respectively. The most common used PCNL tract size was 22 Fr (64.3% of the cases). A total of seven patients presented with renal abnormalities: three patients with horseshoe kidneys, two patients with kidney malrotation and two patients with a duplicated pelvicaliceal system.

**Table 1:** Patients demographics, stone, and perioperative parameters

Number of cases	*n* = 33
*Patient demographics*	
Age (years), median (IQR)	54 (49–67)
Gender, *n* (%)	
Male	16 (48.5)
Female	17 (51.5)
BMI (kg/m^2^), median (IQR)	25.6 (23.2–29.0)
*Stone characteristics*	
Total stone size (mm), median IQR	35.0 (28.5–43.5)
Complete or partial staghorn (yes), *n* (%)	20 (60.1)
*Operative data*	
Operative time (min), median (IQR)	47 (36–65)
Access number, *n* (%)	
One	26 (78.8)
Two	5 (15.2)
Three	2 (6.1)
Access size, *n* (%)*	
30Fr	15 (35.7)
22 Fr	27 (64.3)
Abnormality type, *n* (%)	
Horseshoe kidney	3 (9.1)
Malrotation	2 (6.1)
Duplicated	2 (6.1)
*Outcomes*	
Stone-free rate after first PCNL, *n* (%)	
No	5 (15.2)
Yes	28 (84.8)
Final stone-free rate, *n* (%)	
No	3 (9.1)
Yes	30 (90.9)
Second look PCNL, *n* (%)	
No	31 (93.9)
Yes	2 (6.1)
Mean hospital stay (days), median (IQR)	3 (2–3)
Mean hemoglobin loss (gr/dL), median (IQR)	1.2 (1.1–1.4)
Complications, *n* (%)	3 (9.1)
Fever, Grade II	2 (6.1)
Bleeding, Grade II	1 (3.0)

Numerical values are presented as median and interquartile range

*The calculation was performed from a total number of access *N* = 42

Treatment outcomes are summarized in Table 1. Primary SFR was 84.8%. Two patients required a second look PCNL. Overall SFR was 90.9%. Median hospital stay was 3 (IQR 2–3) days. Mean hemoglobin loss was 1.2 (IQR 1.1–1.4) gr/dL. Complication rate was 9.1%, all Grade II complications. Two patients had postoperative transient fever and one patient had bleeding that resolved conservatively.

## Discussion

In this study, we evaluated the effectiveness and safety of nonpapillary prone ECIRS in an unselected cohort of patients. Including a total of 33 patients, a final SFR of 90.9% following the procedure was observed. Second look PCNL was required in two patients with staghorn stones. Only 3 complications were reported all of them being ≤ 2 Grade according to the Clavien–Dindo classification. With the median hemoglobin decrease of 1.2 g/dL, one patient had continuous bleeding prolonging the hospital stay. These findings are in accordance with other series [11] and attest to the effectiveness and safety of the procedure.

**Fig. 2:**
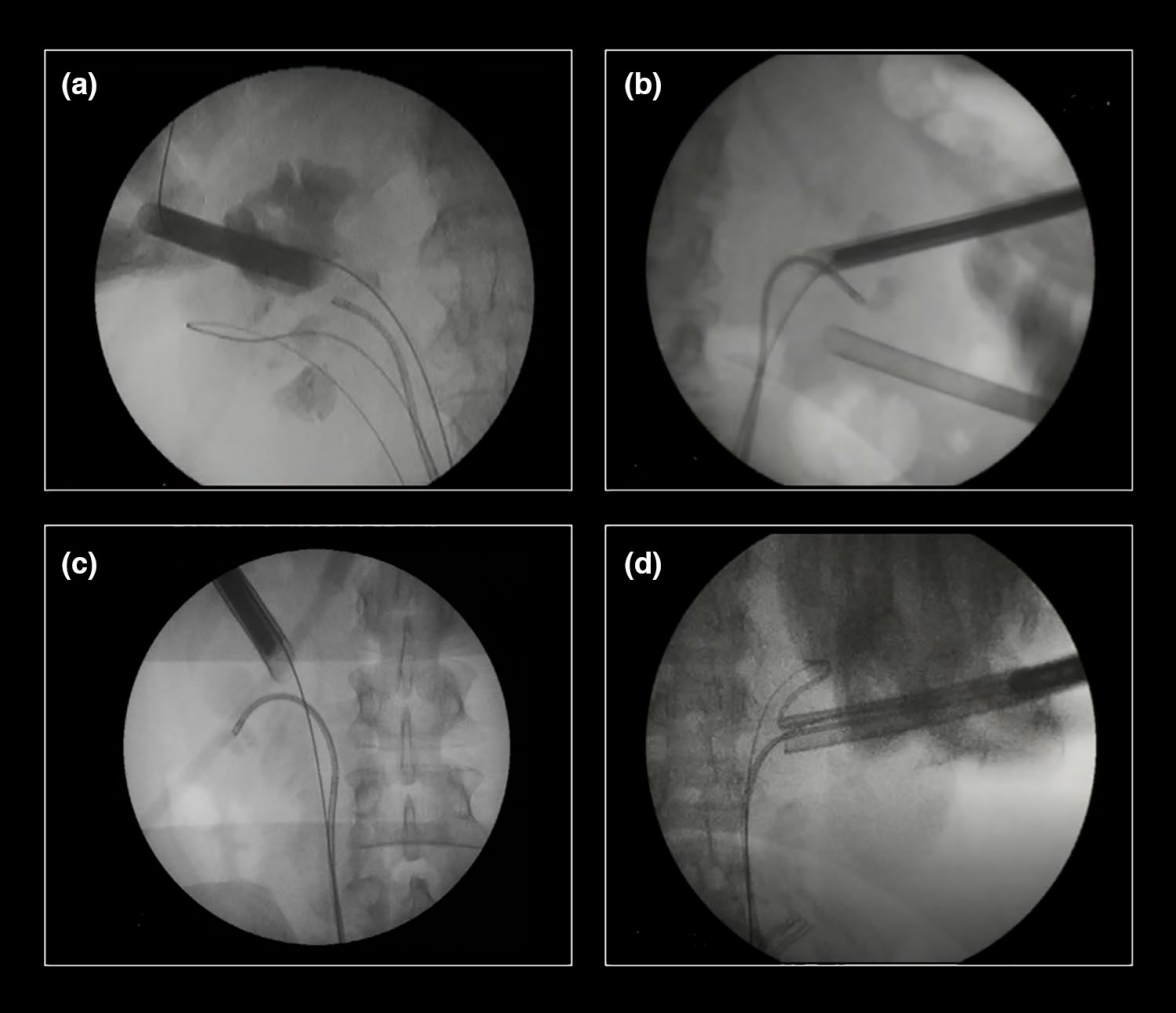
**a** In a duplicated pyelocaliceal system, the upper pole was approached by PCNL and the lower pole by fURS; **b**, **c** Show the flexible scope in calyces not accessible by the nephroscope; **d** Encrusted stent being retrieved from the anterior access sheath

ECIRS is an effective procedure for complex stone treatment [3]. In a way, it has been viewed as a natural evolution of PCNL [4]. ECIRS achieves higher SFRs with fewer complication rates when compared with PCNL [3, 5, 7, 15, 16]. Adding fURS to the PCNL procedure is both a diagnostic and therapeutic advantage [2]. From the diagnostic perspective, fURS allows evaluation of the anatomy of both the lower and upper urinary tract [2], endoscopic control of the renal access [7, 9, 17] and final endoscopic assessment for residual fragments [2, 18, 19]. From the therapeutic perspective, fURS allows the possibility of treating ureteral stones [20], treating contralateral kidney stones [21, 22] and can be a valuable tool in synergistic cooperation during renal stone management [18]. The fURS is of determinant value in accessing stones in calyces not reachable with the nephroscope (see Fig. 2), with the possibility of complete laser fragmentation or relocation of those stones to be extracted by the antegrade access sheath, a maneuver that has become known as “pass the ball” [23]. In our perspective, this is the main advantage and one of the main indications for the ECIRS. Whenever patient anatomy poses difficult angles to PCNL or there is a renal abnormality, ECIRS is truly a game changer. In our study, we report two cases of kidneys with the duplicated systems (see Fig. 2), two cases of kidney malrotation and three cases of horseshoe kidney. In these cases, the use of fURS prevented the need for additional punctures and the possibility of using maneuvers like “passing the ball” significantly facilitated the procedure. Furthermore, ECIRS can be crucial whenever additional access represents a great risk. The latter is of particular importance for stones in upper calyces located over the 11th rib [24]. If one can eliminate the need for multiple punctures and, especially, excessive torque with nephroscope to reach difficult calyces the risk of bleeding is reduced [3, 5]. We are convinced this is the main factor for the low hemoglobin loss that we report in this study, which is also consistently reported by many groups [5, 7, 15, 16].

Although conventional PCNL is more widely performed in a prone position, the first report of ECIRS was in the GMSV position [1]. Thereafter, there were several other reports of ECIRS in the prone split-leg position [5, 7, 9, 25]. The GMSV position has been proposed to have some anesthesiologic advantages, allow better drainage of lithotripsy fragments and avoid the need for patient repositioning [2, 3, 18]. On the other hand, the prone split-leg position provides a wider working space, less mobility to the kidney, less pelvicalyceal system collapsibility, shorter tract length, and possibility for gaining easier upper pole accesses [2, 8, 18, 19]. Hamamoto et al. recently compared the two positions in a multi-institutional analysis [26]. They found equal stone-free status, longer operative time in GMSV position, but with lower infectious complications and urinary tract injuries. Similarly, Perella et al. found comparable outcomes for both prone and supine positioning in PCNL. Supine position was also associated with lower high-grade complication rates [27]. The downwardly orientated access sheath could have provided better drainage and prevented high intrarenal pressures, thus lowering infections in supine position [28]. Nonetheless, our results do not show high urinary infections rates nor urinary tract injuries.

We believe that our positive results mainly arise from the standardization of the procedure. Our antegrade access of ECIRS procedure follows the same rules and steps of that of PCNL tract establishment, which have been described previously [10]. There are some key tips and tricks that we think truly impact the outcomes.

**Fig. 3:**
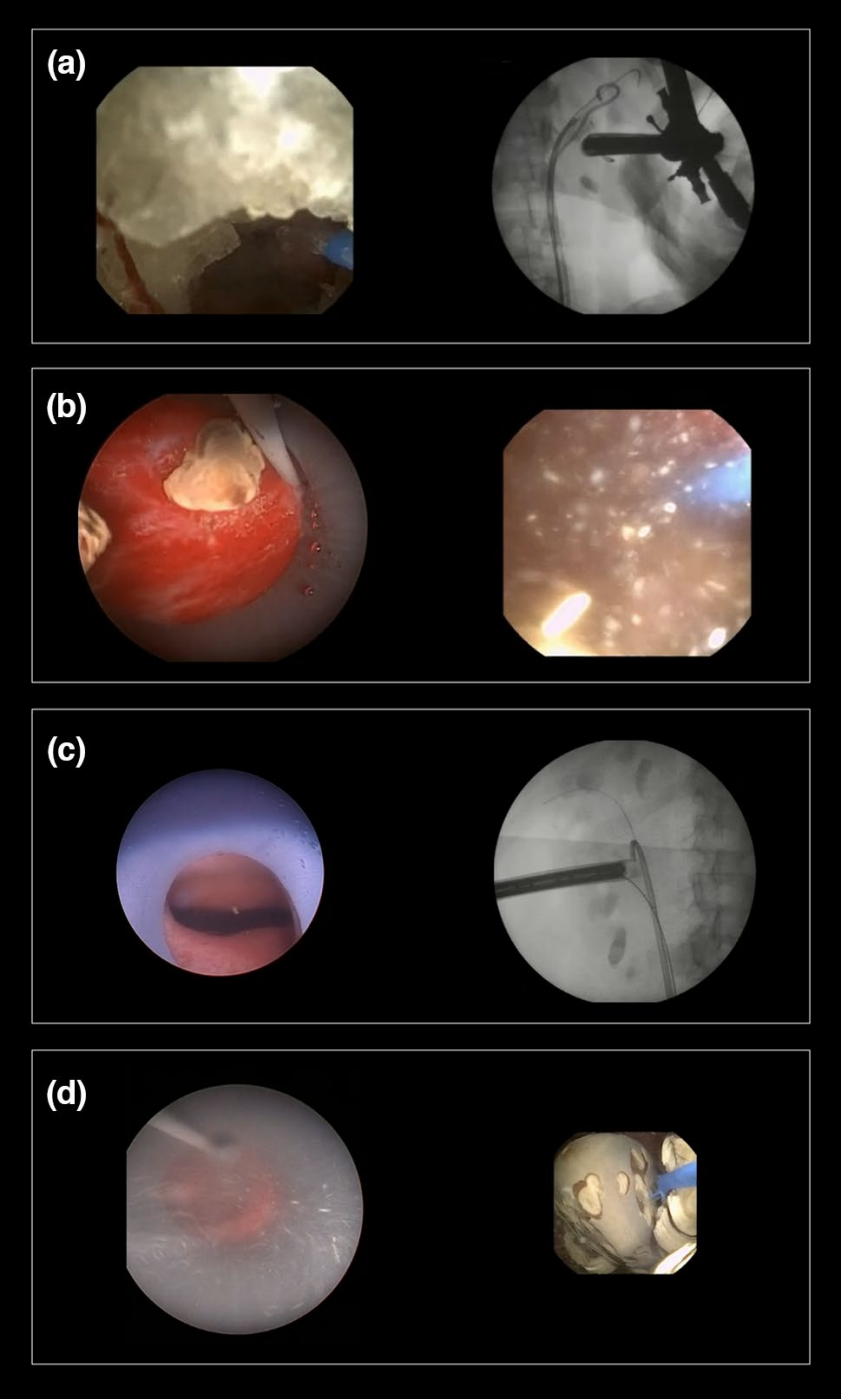
**a** Proximal end of an encrusted stent being released with fURS and assisted with PCNL; **b** Stone entrapment by antegrade access sheath while fURS lithotripsy; **c** flexible scope is seen through the nephroscope that should be retrieved inside the sheath, while performing the fURS; **d** Dust coming out the antegrade access sheath from the fURS lithotripsy

First, we prefer the prone split-leg position due to the above-mentioned advantages (see Fig. 1). Secondly, the medial nonpapillary puncture allows increased maneuverability inside the pelvicalyceal system and access to almost every caliceal group [29]. We believe that this access also reduces parenchyma disruption when moving the nephroscope and thus prevents urinary lesions and bleeding. Third, having a guidewire “through and through”, before any further action, for us is a critical safety step, in which retrograde fURS can be helpful to achieve [30]. Fourth, a 22Fr access sheath may be the optimal size to perform ECIRS in most cases. It possesses the appropriate diameter to treat large stones, has the possibility to incorporate small probes of dual-energy lithotripters with integrated suction and its smaller size allows more mobility inside the pyelocaliceal system when compared to 30 Fr standard access. Fifth, the difference between access sheath and nephroscope should be 4Fr [3] to maintain a low-pressure system and decrease the risk for infectious complications. Sixth, we favor the use of an UAS for the retrograde access, as it clearly facilitates the ureteroscope insertion and retrieval, especially in the prone position. Moreover, when both nephroscope and ureteroscope are inserted, using an UAS improves saline flow and helps to keep intrarenal pressures low. Seventh, with the high flow provided by the dual irrigation system, one can use high-power settings in laser lithotripsy (up to 60 W) without the risk of increasing intrarenal temperatures. We consider a “self-popping” technique [13] more useful than dusting, because one can evacuate bigger fragments through the antegrade access sheath. Eighth, unless working in completely duplicated collecting systems, the activation of the lithotripter from the percutaneous tract should be avoided at the time of RIRS, due to the risk of damaging the flexible ureterorenoscope (see Fig. 3). However, if the lithotripter has an integrated suction, it can be useful to activate it inside the PCNL tract sheath in order to aspirate fragments generated with laser lithotripsy. Nineth, the PCNL sheath should be considered as dynamic tool which can be helpful in trapping stones. Whenever the access sheath “catches” a stone, closing antegrade and opening retrograde irrigation can facilitate faster evacuation of the stone (see Fig. 3). Again, a lithotripter with incorporated suction can also help in this case. In this aspect, GMSV positioning could favor easier stone extraction due to gravity advantage. However, in our experience, stone evacuation in prone is also effective when combining retrograde irrigation and antegrade aspiration. An additional advantage of using fURS is the prevention of stone migration to the ureter. Finally, in some specific cases, certain adaptive variations are necessary. For instance, in cases of encrusted ureteral stents, it is useful to release it first by fURS and extract it from the antegrade access (see Figs. 2 and 3). Likewise, a meticulous preoperative planning of the procedure with the possibility of a 2-step procedure should be considered for complete staghorn stones. In our series, we aimed to clear the pelvis and one of the calyces in the first procedure and approach the remaining in a second intervention.

The number of patients included in the might be considered as a limitation. However, the unselected nature of this cohort strengthens the findings of the effectiveness and safety of the procedure. The procedures were also performed by experienced surgeons, which could lead to the overcorrection of some surgical steps potentially impacting the study outcomes. The technique employed was with a prone split-leg position using a nonpapillary puncture with Amplatz dilators. The given limits generalization of our results to other patients’ population. Furthermore, stone composition was not analyzed. Finally, the SFR evaluation was mainly performed with KUB and ultrasonography which could have missed some residual stones.

## Conclusion

Nonpapillary prone ECIRS is an effective and safe procedure. We obtained a SFR 90.9% with only 9.1% of low-grade complications. Standardization of ECIRS procedure is critical to achieve good outcomes. Patients who benefit the most are probably the ones where additional punctures can be avoided by the use of this technique, namely patients with renal abnormalities, incrusted ureteral stents and staghorn stones.
